# A dual perception of an ageing orofacial appearance— an interview study

**DOI:** 10.1080/17482631.2025.2516618

**Published:** 2025-06-08

**Authors:** Sara Henricsson, Nina Lundegren, Viveca Wallin Bengtsson, Pia Andersson

**Affiliations:** a Faculty of Health Sciences, Kristianstad University, Kristianstad, Sweden; b Faculty of Odontology, Malmö University, Malmö, Sweden

**Keywords:** Aging, older adults, oral health, orofacial appearance, self-perception

## Abstract

**Objective:**

This study aimed to explore how people aged 65 years and older experience their orofacial appearance (OA).

**Method:**

Semi-structured interviews were conducted with 20 strategically recruited participants aged 65–79 years from the Swedish National Study on Aging and Care—Blekinge (SNAC-B) in Karlskrona. A semi-structured interview guide was developed, and the interviews were analysed using thematic analysis to identify patterns in the data.

**Results:**

The older adults’ experience of their OA was represented in four themes: (i) you kind of have to like the situation as it is now—to accept orofacial appearance in its current state; (ii) an ageing orofacial appearance—a slow continuous downhill slope; (iii) looking good for others to fit the social norm; and (iv) keeping up orofacial appearance—seemingly without effort.

**Conclusion:**

The older adults in this study had a dual perception in relation to their own OA. Although society often values a younger looking appearance, striving for a youthful appearance is seen negatively, which may potentially reflect the complexities of the perceptions of one’s own ageing OA. Only those with dental issues found the impact of teeth on OA perception to be particularly significant.

## Introduction

Orofacial appearance (OA) has been suggested to be of great importance for people of different ages (Müller et al., 2017). Although older adults today, compared to previous generations, are a more educated group (Henricsson, et al., 2024; Rydberg Sterner et al., 2019), have higher cognitive function, and often work well beyond retirement age (Rydberg Sterner et al., 2019), OA is no longer considered a priority for this group (Neumann et al., 1989; Vallittu et al., 1996).

Appearance refers to how someone or something looks or appears visually. It includes physical attributes, such as height, weight, and facial features (Appearance, 2024). Different terms are used in relation to OA to describe the appearance of the face and oral region, such as facial and dental appearance. Dental appearance has been described as an important aspect of physical appearance, which can have a significant impact on a person’s self-image, social interactions, and overall quality of life (Eli et al., 2001; Müller et al., 2017). Facial and dental appearance are often the first attributes to be assessed by others in social interactions (Duvernay et al., 2014). However, there is no consensus on the definitions and concepts.

How people perceive their appearance depends on several aspects and is based on more than what is visible. Body image is one example and is defined by how people perceive their physicality, their physical appearance in particular, and involves perceptions of appearance-related experiences and attitudes, including feelings, beliefs, thoughts, and behaviours (Cash, 2012). In other words, perceptions of self-perceived oral health problems or the aesthetic appearance of teeth may influence how a person perceives their OA. Building on body image and physical appearance as presented in Cash, (2012) and the orofacial aesthetic scale (Larsson et al., 2010), OA has been defined as the individual perception of one’s physical characteristics of the face, mouth, and teeth.

With increasing age, normal age-related changes occur in the OA region, which can affect how OA may be experienced. Visible ageing of the face is a complex three-dimensional process that includes remodelling of the facial skeleton, soft tissue changes involving redistribution and loss of subcutaneous fat (Coleman & Grover, 2006; Mendelson & Wong, 2012; Swift et al., 2021), and skin changes (Coleman & Grover, 2006; Swift et al., 2021). Each of these anatomical structures undergoes its own ageing process, but they are also interdependent. Consequently, a change within one structure affects another (Swift et al., 2021), contributing to the appearance of an ageing face and its expression (Mendelson & Wong, 2012; Swift et al., 2021). Obvious age-related changes include facial wrinkles, lack of firmness, and yellowish skin colour. Visible age-related changes in the mouth and teeth include elongation of the upper lip (Desai et al., 2009; Iblher et al., 2012), reduction in lip volume thickness (Iblher et al., 2012), darkening of tooth colour, discoloration of fractures and cracks along the enamel surface, wear and chipping of the enamel, and dentin exposure (Algarni et al., 2018; Lamster et al., 2016).

The oral health status can also change with age. These changes may have a major impact on the perception of OA. Epidemiological research suggests that individuals aged 68–76 years have better oral health than previous generations (Critén et al., 2022; Diep et al., 2023; Suominen et al., 2018), with more teeth remaining and a decreased occurrence of oral diseases. Regardless of oral health status, the perception of one’s oral health and/or aesthetic appearance may influence the perception of OA (Larsson et al., 2021). Several studies have reported on the ageing of orofacial features in older adults’ (He et al., 2021; Iblher et al., 2012; Mendelson & Wong, 2012; Swift et al., 2021) or addressed body image in older adults (Bhatia & Singh, 2021; Clarke & Griffin, 2008; Hurd, 2000; Krekula, 2016), especially that of women. However, the perspectives of older adults themselves on their ageing orofacial features are, to our knowledge, underrepresented in the research. It is therefore of interest to explore how this group of more educated older adults encountered in dental care today perceives their OA, considering that they may have different demands and expectations regarding their oral health and OA.

## Aim

This study aimed to explore how people aged 65 years and older experience their orofacial appearance (OA).

## Methods

### Design

To address the aim, this study employed an exploratory qualitative design using strategic sampling based on semi-structured interviews. An inductive thematic analysis, adhering to the guidelines established by Braun and Clarke, (2022), was used to analyse the data. The study adheres to the checklist of the Consolidated Criteria for Reporting Qualitative Research (COREQ) (Tong et al., 2007).

### Recruitment and participants

Participants were recruited from the SNAC-B study in Karlskrona, southeastern Sweden, one of four research centres in the ongoing population-based longitudinal cohort study of the Swedish National Study on Aging and Care (SNAC). SNAC, initiated in 2001, aims to increase knowledge of ageing, with the possibility of studying ageing from the transition from work to retirement and older age (Lagergren et al., 2004). Invited participants are re-examined every six years until the age of 78 years and every three years thereafter. The SNAC-B study has been described in more detail elsewhere (Henricsson, et al., 2024; Henricsson, et al., 2024).

Twenty participants were strategically recruited from the SNAC-B registry follow-up 2019–2021, covering a variety of ages, sexes, and numbers of teeth to ensure heterogeneity and diversity. The first five women and the first five men with the most natural teeth and the five women and five men with the least number of teeth were selected from a coded list of participants aged 66 and 72 years at the 2019–2021 SNAC-B follow-up. To be included in the study, participants needed to understand and speak Swedish fluently, be of retirement age, and fall within the age range of 65–79 years. The participants will henceforth be referred to as “informants”.

Informants received detailed information about the study’s aim by mail and were contacted by the first author (SH) via phone within one week, with a request to participate. Out of 20 the informants, four declined to participate, and one was unable to attend. Therefore, mail was sent to five additional individuals who were contacted in accordance with the previous procedure. In total, 25 informants were invited, of whom 20 agreed to participate: ten men and ten women. The average age of the informants was 72 years (68–76 years) (Table 1).

**Table 1.** Description of included informants.

ID:	Gender	Age
I:1	Male	68
I:2	Female	68
I:3	Female	74
I:4	Female	69
I:5	Female	68
I:6	Male	68
I:7	Male	76
I:8	Male	74
I:9	Female	76
I:10	Female	69
I:11	Male	74
I:12	Female	76
I:13	Female	76
I:14	Male	76
I:15	Male	68
I:16	Male	68
I:17	Female	69
I:18	Male	74
I:19	Male	69
I:20	Female	74

### Data collection and procedure

Individual interviews were conducted between May and June 2023 at the Blekinge Institute of Technology Research Clinic. Prior to the interviews, informants were asked to approve the use of electronic recording equipment.

A semi-structured interview guide was developed and used during the interviews (see Supplementary File). A pilot interview was conducted, after which one question was rephrased, as it was perceived as unclear. The interview guide addressed questions regarding the importance of OA. For example, “Can you tell me what your thoughts are concerning your orofacial appearance?”. If necessary, probing questions were asked by informants to clarify and enrich the descriptions or deepen their answers. Examples of probing questions were, “Could you give an example?” or “Could you explain that further?”.

**Figure 1.**
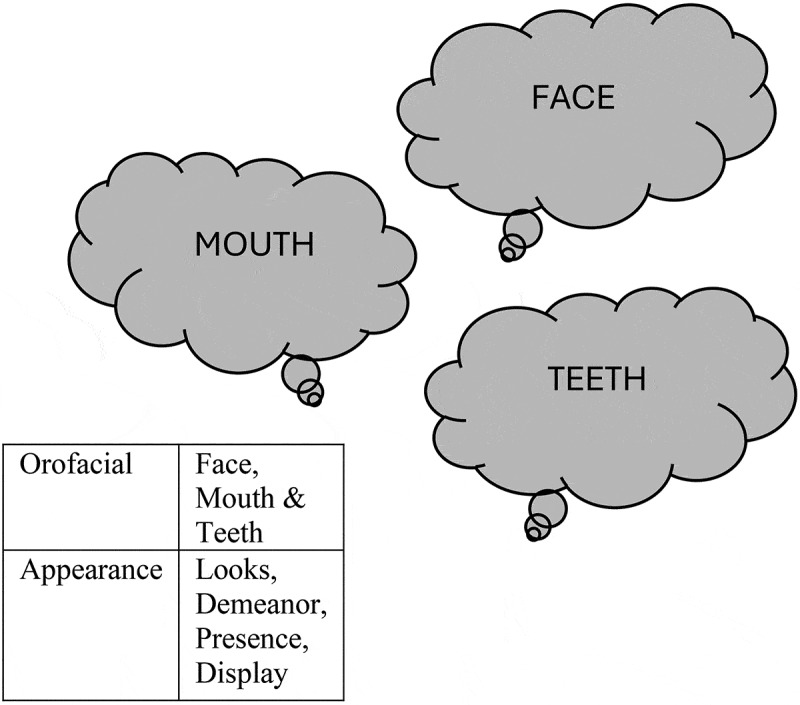
The individual perception of one’s own physical characteristics of the face, mouth, and teeth.

Before the interview began, informants were told that the aim of the interview was for them to share their experiences and attitudes towards OA. This was done to facilitate and help informants reflect on their experience of OA. A document with the definition of OA used in this study and synonyms for the word “appearance” (Figure 1) was placed in front of them during the interview. All interviews were conducted by the first author (SH), with the support of the last author (PA). Neither had any personal relationship with the informants. On average, the interviews lasted 27 minutes (ranging from 15 to 48 minutes) and were transcribed verbatim. This interview study was part of a larger interview study with an average duration of 58 minutes.

### Data analysis

Thematic analysis, a method for analysing and interpreting qualitative data (Braun & Clarke, 2022), was guided by the research question: How do older adults experience their OA? The data analysis process followed the six phases in accordance with the process outlined by Braun & Clarke, (2006, 2022). The first phase, (1) *familiarization with the data and writing familiarization notes* involved several re-readings, annotations of initially emerging patterns and a comprehensive review of the interviews by all authors (SH, PA, NL, VWB), followed by a joint discussion. During the next phase, (2) *systematic data coding*, the first author (SH) conducted the initial coding and discussed the codes with the last author (PA). The codes were then jointly discussed by all authors. In the third phase, (3) *generating initial themes from coded and collated data*, the first author combined codes with common features into potential themes and discussed them with all the authors. The fourth phase, (4) *developing and reviewing themes*, was preceded by individual rereading of codes and a review of the initial themes by all authors to further develop the themes. In the fifth phase, (5) the relationships between codes and between themes were reviewed by the authors to *refine, define, and name themes*. In the final phase, (6) the first author organized the collated data into a narrative which resulted in the *writing of the report* with selected representative quotes for each theme (Braun & Clarke, 2022). Data were organized and processed using NVivo Release 1.7.1 (QSR International Pty Ltd.). Examples of the identification of codes and themes are listed in Table 2.

**Table 2.** Examples of codes and themes.

Citation	Code	Theme
*“… of course you want to look better. Of course you do. But importance and importance …. It… you kind of look the way you do, you have to accept that” (I:13)*	Acceptance of own orofacial appearance	You kind of have to like the situation as it is now—to accept orofacial appearance in its current state
*“… yes, of course you think that you’re getting older when you see yourself and look more and more like your mum. It’s just that, well, it goes too fast, I think. I often think like that” (I:12)*	Physical change that occurs with age and the feelings around it	An ageing orofacial appearance—a slow continuous downhill slope

### Ethical approval

All procedures were conducted in compliance with the established ethical guidelines of the World Medical Association Declarations of Helsinki (Bibbins-Domingo et al., 2025). An amendment application to the ongoing and previously approved SNAC study (No: LU 604/00) was approved by the Swedish Ethical Review Authority (No: 2023–01020–02), which has been reviewed by the same department that reviewed and decided on the previously approved application. Informed consent, both verbal and written, was obtained from each informant before the interviews. Data were anonymized and securely stored to protect the interests of study informants (Regulation (EU) 2016/679 of the European Parliament, 2016). Informants were informed that their participation was voluntary and that they could withdraw from the study at any time without consequences.

## Results

The thematic analysis of how older adults’ experience their OA generated four themes: *you kind of have to like the situation as it is now* – *to accept orofacial appearance in its current state; an ageing orofacial appearance* – *a slow, continuous downhill slope; looking good for others to fit the social norm;* and *keeping up orofacial appearance* – *seemingly without effort.*

### You kind of have to like the situation as it is now – to accept orofacial appearance in its current state

The first spontaneous thought expressed was that OA was not something that the informants had thought about or reflected upon. Some immediately commented on the insignificance of OA, and instead of reflecting upon OA in relation to themselves, they evaded a discussion of the topic. It was easier to dismiss or joke about it. Occasionally, the informants would acknowledge a perceived problem with OA but would then swiftly diminish its significance by cracking a joke. Others emphasized that they had fully embraced OA in its current state and did not consider it to be as important as it once was. Over time, they had grown more accepting and content with their appearance, and a few women mentioned that they no longer felt the need for makeup. Other people’s opinions no longer had the same impact. They believed that it might have been more important to them earlier in life if their OA had been subject to the opinions of others.

I find it a bit difficult to talk about, because I haven’t considered it. Neither my face nor my teeth (I:18)

Well, you look like you do. You can’t change it. I never use makeup anymore. Perhaps I should, but I don’t (I:13)

Despite accepting OA in its current state, the informants reflected that OA had changed and was now different from earlier in life, a realization they in no way found to be burdensome. They assumed that this was because they now had different priorities and with age comes a certain maturity or self-awareness, along with a kind of basic security in not having to take yourself too seriously. They were quite content with their OA, recognizing the flaws that had emerged as a result of life and all that it entailed, but felt no urge to rectify them. As one informant said, *“it doesn’t make you feel better, it’s just bothersome” (I:3).*

Talking about their own OA proved to be a challenge for some informants, as it was not something they usually discussed with anyone. The same was true for those who expressed that they had been fortunate enough to look good. However, for them, it was more about not having to think about their OA, as it had never been a source of concern.

Nah… But it feels a bit silly to say it, but I looked really good when I was young.//. But I’ve never really thought about it myself, like until someone says to me or mentions it. (I:20)

### An ageing orofacial appearance – a slow, continuous downhill slope

How the informants viewed their OA in relation to their age differed depending on how they perceived their age. Despite emphasizing the importance of an OA aligned with their ageing, they frequently made comparisons with others in their age. They had the impression that others their age had aged and looked visibly older, and although they did not want this to be the case, they realized that they probably had, too. However, some only became aware of these changes when they looked at photographs from a few years ago. The development of facial wrinkles was the most apparent physical change in OA, which they linked to the process of ageing. They also observed that their teeth had become more yellow and changed position. The change in OA was less apparent for those who expressed being actively engaged in all phases of life. In contrast, the other informants tangibly experienced the physical changes in OA with advancing age.

…my God, how old they have got … … . then you think… do I look just as old myself – I guess I must do – like, nooo, you know (laughs). It’s a bit like that … so, sure, it’s a little reminder that it probably applies to you too. (I:20)

Sometimes you think to yourself, my goodness, those teeth are really yellow. (I:3)

Less pleasant physical changes in OA that occur with age include increased facial hair growth, nasal growth, and the perception of facial skin being looser and sagging. The women emphasized the effect of ageing on their facial features and appearance and that remembering to smile was important to avoid looking angry because of the down-turned corners of the mouth. One thing was knowing that OA changes with age, while experiencing it was a different matter. They disliked looking older, and most of them felt younger, but they also believed they looked more youthful than their peers and wanted to be perceived by others as younger looking. It was okay to age, but should preferably not be visible. The change in OA left some female informants feeling a sense of loss, like looking at yourself from the outside without the ability to intervene.

But now that I think about it, something that really bothers me is that I get so many whiskers.//. that I have to pull out all the time. It is very irritating. As it is, if there is one … oh no, so you get a fairly long one. It happens fast and around the mouth and so on. Coarse, really tough. It is no fun. (I:4)

Yes, of course I can see that… Good lord. Stop. I can not take it any more … //. It is the fact that you change. Your appearance, wrinkles and, well, structure. Everything. You … well, that is how it is … and it is not always that bloody easy to deal with. It … well, I had not really considered it (I:12)

The shift in the perception of OA, caused by diseases or medical conditions, also brought about a feeling of being an observer of the changes in appearance. The informants who faced issues in some sense—periodontal disease in particular—noticed changes in their OA only in relation to that problem. Some of the informants currently faced significant issues, and they experienced that the change in OA caused by periodontitis contributed to an aged OA, making them look older than they were. Despite different views on the importance of OA, all informants acknowledged the importance of teeth in relation to OA, as most had or have had oral health problems in one way or another.

…when I lost my teeth, I looked completely different to myself.//. that. you look a little too old. Even though I do not feel old inside. So it is a big minus if you have a prosthesis or no teeth… like me down there… none (I:2)

### Looking good for others to fit the social norm

Despite the initial downplaying of the significance of OA, most acknowledged that it was important to some extent, considering that the face, mouth, and teeth are prominent and the first thing that others notice. However, opinions were divided regarding the extent to which OA was important. For some informants, it was crucial that they were perceived to have a pleasant appearance. Others were careful not to exaggerate the importance of OA but to still give a pleasant impression and look “normal”. Informants who attached importance to what others attributed to their OA sought validation from others and spoke of receiving compliments on their youthful appearance as being particularly important.

But this… it is probably highly significant because that is the first thing people look at. So, well, it is probably pretty significant, but I guess there is not much you can do about it. Or, well, obviously you can. But if you do not want to, I guess you will just have to look the way you do. (I:4)

Yes, of course, the face is really important. It is the first thing you encounter when you meet another person.//. I mean, I am not overly concerned by it. I mean, it is important to some extent, it is, but as I said, I want to look healthy. (I:5)

I think teeth are actually really important… It looks dreadful… when some people turn up with… big gaps in it, it… you react to it. You cannot help it. It is probably… it is probably the most important actually… .//. Yes, at least what you see from the front when you laugh and so on. (I:11)

OA was considered important in social contexts and in relation to other people, albeit in different ways. The efforts put into OA varied based on the situation and the individuals involved. For some informants, less effort was made on OA when it came to their immediate family and friends, as they already knew you well, while for others, it was just the opposite. It was more important to take care of their OA, since their families meant the most to them.

You do not think… about it when you meet close friends and family and so on, and children and that, then you do not give a second thought to what you look like. But, obviously, if you are going to meet… I meet an awful lot of people. I am politically active, so I meet a great many people and then obviously you think about how you look. About trying to fix what can be fixed, so to speak. So that is what you do (I:20)

Although the informants themselves had accepted the way their OA looked now, they felt it was not acceptable based on the society’s view of OA. It was expressed that appearance has become highly significant in society because of the pressure to conform to certain standards, making them believe that an ageing face and poor dental health impact one’s perception of others. Opinions differed on whether they were affected by these societal standards or trends. Some informants believed that it did not affect them at all, while others believed it absolutely affected them. In addition, some felt a tinge of annoyance or jealousy towards others, informants who still had something they themselves lacked or had lost in their OA.

Or, well, obviously you can. That I do not exactly have particularly lovely teeth… I am sure it affects how people see [me]… even if they do not express it, so to speak, I assume that they notice that kind of thing. If nothing else, I am sure those close to me do, so to speak, you know… And then it is reasonable to assume that if you meet people you have not seen for a while, they might also notice.” (I:8)

It is obvious when you see all those people on TV and so on with beautiful white, lovely teeth. I think they probably have more than 32 (laughs). But they… well, like… Then you think about it. Ugh, I only have so and so many teeth… (I:7)

Well, that is how it is. So, it is…. I do not really care about trends. Hardly at all. When it comes to that…//. Well, it just needs to look fresh and neat then, like, it does not matter what the current trend is. If, these days, it has to be gleaming white when you open your mouth or so on. For it to see normal out, I mean. (I:1)

The informants found it difficult to cope with the challenges brought on by non—age-related normal physical changes in OA that were somehow a concern for them. They felt that they could not conceal the problem, but instead that it was visible to others. These changes were perceived to affect social interactions, particularly if they had damaged teeth, periodontitis, or while awaiting tooth replacement. The affected informants refrained from smiling or opening their mouths in social settings. In a few cases, bad teeth or lack of teeth had a major impact on their social life and contributed to involuntary loneliness through self-imposed isolation.

No, no way. No. Sitting close to strangers. That they might… no. No. No way …//… Well, I cannot speak normally. I cannot meet… I am so embarrassed…//… I am embarrassed by my speech, appearance and that I spit when I talk. With or without teeth, it is so awkward; plus that it smells. (I:2)

### Keeping up orofacial appearance – seemingly without effort

Whatever the reason, it was important for the informants to keep themselves well-groomed and take care of themselves, such as trimming their eyebrows and other facial hair growth in an attempt to delay or postpone the appearance of ageing. Most of the female informants were keen to look after their OA so as not to look older than necessary and to do what they could to slow down the changes brought about by ageing. Many of them found pleasure in applying makeup, whereas others chose stylish glasses or brushed their teeth with whitening toothpaste. Conversely, the male informants stressed that their daily grooming routine, which included brushing their teeth, washing, and shaving, remained unchanged, regardless of social occasions or the people they would encounter. While some felt it was important to take care of themselves to keep up their appearance, others were careful to insist that they did not care excessively about their appearance or that it was a matter of vanity. It was something else because, *“men don’t go and look in the mirror” (I:14)*.

I mean, I think it is important. I do think so. Erm… So you do not decay any more than you do anyway as you get older (I:17)

…but, on the other hand, I have … this thing about aging. A little hair growing here and there. Eyebrows like bushes. I keep an eye on that kind of thing. I do…//… So, that sort of thing, you have to groom yourself, like trim a bit, like to hold, as I say, hold it back a bit. (I:1)

Taking care of their OA, whether this meant brushing their teeth, shaving, or applying make-up, impacted both OA and their general well-being. Looking decent was important for one’s well-being and to leave a favourable impression on others. In this context, informants stressed the importance of taking care of their teeth for a healthy appearance, which became especially evident for those with damaged or missing teeth.

But teeth have also been a part of me so, naturally, I would like to replace them… .//. I do not know. But it has been very important to me. So obviously I have invested in it. But it… in terms of both appearance and function. Of course, while I was waiting for the implant to arrive, I was aware what it means for the mouth. (I:5)

Apart from various dental treatments, such as the installation of implants, most informants stated that they would never undergo surgery to change or improve their OA. However, several of them had an idea of what they would like to “fix” in their OA if they could wish, such as lifting their chin and upper eyelids, which they found to be drooping, or whitening their teeth. Those who faced issues with their teeth desired permanent implants over removable dentures.

Possibly teeth whitening. I do not think that would be so bad. But I would not have surgery…//… I think it would be nice if they were a bit whiter. But it is definitely not something that is necessary. That it is not. (I:17)

And then… it is not like I can just stick a prosthesis and my teeth will look whole and good. I want to have good teeth. And if they are not my own, they must be as close as possible to my own. (I:5)

A few informants underwent facial surgery of some kind. The procedures were either medical or cosmetic in nature. Whatever the nature of the procedure used and whatever the reason, it should preferably not be visible that a procedure had been performed. Many informants held strong critical views on aesthetic procedures for cosmetic purposes only. Aesthetic corrections that were medically necessary were, however, found to be more acceptable.

Yes of course it has, because I did [tattooed] a lip liner so my mouth stands out more… without injecting anything into the lips, the lips stand out more…//. But if I had not mentioned it you would not notice. So, that was the aim when we did it, that it would not… be visible…//… Of course, it also makes you look a little sprightlier; at any rate, you feel sprightlier. (I:4)

Medically. Essential things, so to speak. One needs to do it for medical reasons, but I think it is verging on crazy to do it just for cosmetic reasons…//… So, I am against that kind of thing if it is only for aesthetic reasons, so to speak. (I:1)

## Discussion

This study’s core finding was the duality that exists in relation to how this group of older adults experiences their OA. This duality became evident through the contrasts in their narratives across the four themes: *you kind of have to like the situation as it is now* – *to accept orofacial appearance in its current state; an ageing orofacial appearance* – *a slow continuous downhill slope; looking good for others to fit the social norm;* and *keeping up orofacial appearance* – *seemingly without effort*. Essentially, the findings highlight an acceptance of or contentment with OA in its current state. However, even if the OA had changed, it could be difficult to witness, although it should not matter. OA was important in social contexts, and informants very much wanted to look younger and be perceived as having a nice appearance, but also felt that their appearance should seem natural and not be perceived as an obvious attempt to look younger. Although teeth are considered an important feature of OA, they were often overlooked in favour of other facial attributes by those who did not consider their teeth to be of concern.

One finding where the duality became clear was the alleged acceptance and contentment with OA, but at the same time an acknowledgement of the challenge of witnessing the change in oneself from the outside without being able to intervene. The meaning attributed to OA was often articulated as a statement that OA was normal for the age reached and that you have the wrinkles you have. Their expressed acceptance of OA reflected either a sense of contentment or, perhaps, the belief that it was expected that they would no longer find OA important. Previous research on body image suggested that older adults accepted their ageing appearance on some level and reported greater self-acceptance than younger persons (Ojala & Pietilä, 2021; Quittkat et al., 2019). Others, women in particular, may feel detached from their bodies and experience a conflict when their external physical appearance no longer matches the perception of their younger “inner self” (Bennett et al., 2020; Cameron et al., 2019; Clark, 2001). However, the duality of feeling content while also finding a change in appearance due to ageing difficulties has not been mentioned in any of these studies. However, it has been suggested that OA and physical appearance are less important and no longer a priority as we age (Neumann et al., 1989; Vallittu et al., 1996) because factors such as health and physical ability become more crucial (Hurd, 2000; Jankowski et al., 2014). This reduction in importance stems not from a loss of value but rather, as Franzoi & Koehler, (1998) and Åberg et al., (2020) showed, older adults may have a less positive view of their facial attractiveness because of societal beauty standards equating youthfulness with physical attractiveness.

Although content, women placed more importance on their current OA than men, who seemed to value the importance of appearance less than women did. However, OA was expressed as being of greater importance to men when they were younger. This is in concordance with previous findings (Åberg et al., 2020; Öberg & Tornstam, 1999; Quittkat et al., 2019), as is the importance of appearance, which remains stable in women of all ages (Quittkat et al., 2019; Tiggemann & McCourt, 2013). This may result from an externalized perspective, where men are judged based on their performance (Calasanti & King, 2018; Halliwell & Dittmar, 2003), while women are often judged based on their appearance (Aharoni Lir & Ayalon, 2024; Kincaid, 2022). According to Sontag, (1972) and the “double standard of aging”, society holds ageing women to a more critical standard than ageing men, and the loss of youthfulness is perceived as more negative to women. A consequence of the double standard of ageing for women in this study was the feeling of being invisible in a society that values appearance and the pressure to conform to societal norms. Clarke & Griffin, (2008) revealed that women expressed that looking older made them invisible to others, and some referred to the societal stereotype that older adults are seen as having little to no social value.

Another finding in which the duality became clear was in relation to the fact that youth and a youthful appearance are highly valued in society, where informants emphasized that OA should be in line with their own ageing, while also desiring a younger appearance. This identified duality has also been described in other studies (Clarke & Griffin, 2008; Jankowski et al., 2014; Muise & Desmarais, 2010) in relation to the sociocultural pressure of looking age-appropriate while resisting the changes that ageing entails. In an interview study (Jankowski et al., 2014), albeit one on ageing and body image, older adults simultaneously perceived a societal expectation to “age gracefully” and look age-appropriate while resisting appearance changes. The findings show that both men and women desired to appear younger. According to Halliwell & Dittmar, (2003), body image continues to be of concern throughout adulthood for both sexes, but women placed a stronger emphasis on maintaining a youthful appearance. Although this contradicts our finding, it is possible, as Halliwell & Dittmar, (2003) found, that men associate a youthful appearance as a sign of being functionally able, whereas women view a younger-looking appearance as a symbol of their value and attractiveness. It is possible that the men in the current study, like those studied by Ojala et al., (2016), were careful to say that they did nothing out of the ordinary—the normal, so to speak—while most of the women were happy to put on a bit of makeup. According to Clarke & Griffin, (2008), women use makeup and non-invasive treatments to satisfy the perceived pressure from society to look younger and conceal their chronological age. According to Ojala & Pietilä, (2021), it was important to avoid appearing old in the eyes of others. However, the informants in this study not only desired a youthful appearance but also believed they looked younger than their peers. Further, according to Allen et al., (2024), a more youthful appearance than one’s peers was found to be linked to better ageing experiences. In contrast, an older appearance was associated with the opposite. In the current study, taking care of your OA was important to the informants, but the time and effort spent should preferably not be visible. It was more about being age-appropriate while maintaining a youthful appearance. About one-third of the participants reported making an effort to look younger, while nearly twice as many self-reported appearing younger. This discrepancy may reflect the complexities associated with the perception of ageing and appearance (Allen et al., 2024).

Being natural and “down-to-earth” was also considered important for the informants in this study. Ojala et al., (2016) showed that maintaining a natural appearance mattered for both ageing women and men, as it was more socially acceptable to look older than to appear unnatural, thus affecting people’s appearance-related choices. According to Hurd Clarke & Griffin, (2007), a natural appearance means passing as a normal, unaltered, and youthful body rather than being natural, per se. In striving to appear natural, it was emphasized, especially by male informants in this study, that it was not a matter of vanity or caring excessively about one’s appearance. It also became apparent that people who were perceived as being excessively concerned about their appearance were frowned upon. Given the age of the informants, it is not unlikely that this originates from the Law of Jante, a deeply rooted consciousness stemming from Scandinavian customs and habits imprinted in early childhood. This is the idea that you should never think you are special or better than others and should certainly never talk about it (Cappelen & Dahlberg, 2018).

The informants in this study widely agreed upon the importance of teeth, and no other orofacial feature had the same level of agreement. However, although teeth are considered an important part of OA, they were often overlooked in favour of other facial attributes by informants who did not consider their teeth to be problematic. Only individuals who had encountered dental issues, either currently or previously, provided detailed information about their experiences and revealed feelings of shame and social isolation consistent with previous research (Johannsen et al., 2012) which likened tooth extraction to the “amputation” of a body part. The emphasis on oral health-related problems in this study was primarily on the social aspect. According to Johannsen et al., (2012), a poor oral status affects the patients´ daily lives, such as avoiding social interactions in different situations. The informants who suffered from periodontitis and tooth loss experienced themselves as having an aged OA. Dental implants were accepted as tooth replacement, while removable dentures were considered a kind of defeat associated with old age. Tooth loss can be associated with ageing, failure, and neglect, and dentures are seen as markers of old age (Rousseau et al., 2014). Similar to the informants in the study by Johannsen et al., (2012), the informants in this study who underwent implant treatment experienced an improvement in their quality of life, but also in what the teeth meant for OA both functionally and in terms of appearance.

All informants in this study were aware of and had observed age-related changes in their teeth and the oral cavity. Warren et al., (2020) reported that part of “appropriate aging” was to accept changes in the mouth that were merely aesthetic and to balance self-care with a healthy perspective on appearance, which can be linked to the previously discussed duality and importance of looking age-appropriate.

We found no research supporting the possibility that older adults from different cultures hold different views on OA. Thus, this study could be replicated with older adults from diverse cultural backgrounds in future research.

### Strengths and limitations

To ensure that sufficient informational power could be obtained in relation to the interview sample, Braun & Clarke, (2022) recommended using the information power model developed by Malterud et al., (2016). With a broad research aim, less specific participant selection, and a previously not clearly defined topic, 20 informants were deemed sufficient based on the aforementioned information power model (Malterud et al., 2016). In retrospect, the number of informants was appropriate because the topic discussed during the interviews was unfamiliar or perceived as difficult to discuss by the interviewees. It was also relevant in relation to specificity, because although participants were strategically recruited with specific aspects of variation in mind, the specificity of the sample could not be predicted, as it was not directly specific to the aim of the study per se (Malterud et al., 2016). Informants were informed about the subject of the study, but in order to avoid interference with the outcome of the interview in any direction, it was not revealed that the interviewer was a registered dental hygienist. It is possible that more focus would be placed on the teeth if the informants were aware of this. However, this approach should increase the credibility of the results. Informants were required to have reached retirement age and were not older than 80 years at the time of the interview. The upper age limit was determined based on the observation that health changes tend to occur around the age of 80 years (Santoni et al., 2015), and we sought to explore, to the extent possible, the experience of OA before these health changes occurred.

To generate a quality analysis and ensure rigour, consistent and strict adherence was adhered to the criteria outlined in the Consolidated Criteria for Reporting Qualitative Research (COREQ) (Tong et al., 2007). The authors discussed their assumptions and interpretations throughout the analysis. A study’s trustworthiness can be determined by considering the criteria of credibility, transferability, dependability, and confirmability (Shenton, 2004). After each interview, recurrent debriefing sessions were conducted by the first and last author, strengthening the study’s credibility. Transferability was considered by providing descriptions and citations, facilitating others to judge the possible transferability of findings into their specific contexts. To ensure dependability, a description of participants was provided, interviews were conducted using the same interview guide as in each interview, data were collected within a limited timeframe, and the analysis followed Braun and Clarke’s, (2022) step-by-step thematic analysis. The conformability of codes and themes was established through discussions at analytical team meetings, including the researchers’ reflections on the analysis. Confirmability was achieved by providing data extracts of participants’ responses; each quotation was accompanied by a reference to the individual interview from which it was drawn.

## Conclusion

In conclusion, the older adults in this study had a dual perception in relation to their own OA. Although society often values a younger looking appearance, striving for a youthful appearance is seen negatively, which may potentially reflect the complexities of the perceptions of one’s own ageing OA. Only those with dental issues found the impact of teeth on OA perception to be particularly significant.

## Supplementary Material

supplementary file OA.docx

Legends_for_figure_tables_and_supplementary_file.docx

## Data Availability

The data presented in this study are available upon reasonable request from the corresponding author. The data is not publicly available because of privacy or ethical restrictions.
