# Supplementary material for: A dual perception of an ageing orofacial appearance— an interview study
Source: Int J Qual Stud Health Well-being. 2025 Jun 8;20(1):2516618. doi: 10.1080/17482631.2025.2516618 (PMC12147477; doi:10.1080/17482631.2025.2516618)
Supplement: supplementary file OA.docx [file ZQHW_A_2516618_SM6561.docx]

Supplementary file

**Interview guide**

Background questions;

Gender

age,

civil status

Educational level

Living area

Can you tell me:

- How you think about your orofacial appearance
- How important your appearance is to you
- Whether the view of orofacial appearance has changed throughout life
- How you see your orofacial appearance in relation to other people of the same age as you
- Something you have done, if anything, to change your appearance over the years, or if you have had to do something you felt has changed the appearance
- The importance orofacial appearance when socializing with other people
- What you think other people see when they meet you
- If there is anything else about the Orofacial appearance that you think is important?

Examples of probing questions:

- Could you give me an example?

- Could you explain that further?

- In what way has it affected you?
