# Supplementary material for: A dual perception of an ageing orofacial appearance— an interview study
Source: Int J Qual Stud Health Well-being. 2025 Jun 8;20(1):2516618. doi: 10.1080/17482631.2025.2516618 (PMC12147477; doi:10.1080/17482631.2025.2516618)
Supplement: Legends_for_figure_tables_and_supplementary_file.docx [file ZQHW_A_2516618_SM6560.docx]

**Legends for figure, tables and supplementary file**

Figure 1. The individual perception of one's own physical characteristics of the face, mouth,

and teeth.

Table 1. Description of included informants.

Table 2. Examples of codes and themes.

Supplementary file: Interview guide
